# Embracing ambivalence and hesitation: a Ricoeurian perspective on anticipatory choice processes at the end of life

**DOI:** 10.1007/s11019-024-10228-5

**Published:** 2024-10-05

**Authors:** Els van Wijngaarden

**Affiliations:** Department of Anesthesiology, Pain and Palliative Medicine, Radboud University Medical Center, Geert Grooteplein Zuid 10, Nijmegen, 6525 GA the Netherlands

**Keywords:** (end-of-life) choice(s), Decision-making, Anticipation, Paul Ricoeur, Phenomenology, Advance care planning, Advance directives, Older adults

## Abstract

Especially older adults are increasingly stimulated to think about, talk about and record their preferences with regard to future (health)care decisions, preferably in a pro-active manner. In this paper, I analyse these anticipatory choice processes. My goal is twofold: Firstly, to provide a deeper understanding of what it actually means to decide in advance about end-of-life treatments or options. Secondly, to make a theoretical contribution to bioethics and ACP-theories by rethinking the concept of end-of-life choices from a phenomenological viewpoint. To achieve this, I start by presenting a case narrative that elucidates how these anticipatory choices are lived. Secondly, I map out a theoretical framework about choice based on the phenomenology of the will of Paul Ricoeur. Finally, guided by this Ricoeurian framework, I investigate the potential meaning of choice in the context of contemporary advance care planning trajectories. The analysis demonstrates that choice and agency always imply notions of passivity and uncontrollability. It also indicates the significant value of hesitation and ambivalence. Moreover, it highlights the importance of the notion of co-responsibility in the context of anticipated end-of-life choices, and the relevant distinction between a (willed) choice and a wish. To improve care and support regarding end-of-life trajectories and to promote meaningful conversations, it is imperative to integrate these underrated elements more substantially in our theories, language and practical approaches. I conclude by suggesting that, in order to do justice to the real-life complexities, we might even need to revise the notion of advance ‘directives’.

*We are at the same time jailers and prisoners* (Ricoeur 1966, p. 98).

*I submit to the body which I guide* (Ricoeur 1966, p. 276).

*I have a way of choosing my way in a situation which I did not chose* (Ricoeur 1966, p. 368).

## Introduction

Especially older adults should think about, talk about and record their preferences with regard to future (health)care decisions, preferably in a timely and pro-active manner. At least, that is what a growing number of governmental bodies, health policy organisations and patient associations in Western countries are advocating (AMA 2016a, b; Dutch Government 2024; NHS 2022; NIA 2023). On patient forums, for instance, people are provided with clinical information and decision aids that are meant to support them in anticipating various end-of-life scenarios: If you are no longer able to breathe adequately and need a ventilator, would you prefer this option? What about artificial nutrition and hydration; in case you are not able to eat or drink, would you want fluids and nutrients to be delivered to you via a feeding tube or would you prefer to avoid that? After considering and discussing the scenarios, people are encouraged to write down their preferences and decisions in an advance directive, or establish specific orders, such as a do-not-resuscitate, a do-not-intubate or a do-not-hospitalise order (Netherlands Patient Federation 2021; NVVE 2022; Sudore 2013).

Planning ahead for their future (health)care by means of advance directives (ADs) is said to help ensure that people get the medical care they want, prevent unwanted treatment, and, as such, avoid unnecessary suffering (AMA 2016b; Hamilton 2017). Additionally, ADs are designed to guide choices for health professionals and close ones, especially once a person is in the late stages of dementia or terminally ill and no longer able to communicate their wishes. ADs should make it easier for close ones to make (health)care decisions for the person concerned (Hall et al. 2012). Moreover, it is suggested that ‘putting a plan in place’ may relieve close ones of decision-making burdens during moments of crisis or grief (NIA 2023). ADs are also expected to help to reduce confusion or disagreement about the choices people would want their close ones to make on their behalf. It could even help close ones to grieve more easily and feel less burden or guilt after a person has passed away (NIA 2023).

Empirical research has repeatedly shown that, in practice, people themselves (as well as their close ones and health professionals involved) do benefit from planning their future care (Overbeek et al. 2019; Rietjens et al. 2017, 2021), and demonstrates that advance care planning (ACP) can be considered (at least partially) an emancipatory response to medical power. It is also reported that unrepresented patients, as well as patients without ADs, are at higher risk of both undertreatment and overtreatment as their wishes are unknown (Chokshi 2020; Pope et al. 2020). Moreover, they are less likely to be enrolled in a hospice, even when curative options are exhausted (Chokshi 2020).

Simultaneously, however, the increasing emphasis on choice at the end of life also has some significant problematic sides that should be considered (Borgstrom 2015; Morrison 2020; Sedini et al. 2022; van Wijngaarden and Sanders 2022). First, although meaningful conversations with close ones about values and wishes regarding the end of life are considered a crucial part of ACP, in practice this often tends to be limited to ‘choice-talk’ with a focus on medical decisions to be made by an individual, merely informed by cognitive (medical) information and aiming at an outcome-oriented target (Antonides and van Wijngaarden 2024; Mol 2008). Such accentuation of rational, information-driven aspects foregrounds the transactional aspects of end-of-life communication, rather than, for instance, the moral or existential aspects (Sallnow et al. 2022). In fact, the moral and existential aspects of these complex choices may even be overlooked. Choice or preference as a clearly defined, rational category does not do justice to the challenges of the contingent and changing illness journeys that people may face when struggling with serious diseases. In these situations, choice quite often is not a main concern or aspiration of the people involved, nor an experienced reality. On the contrary, the people involved often find themselves in one of the most unsettling life experiences in which they – for a wide variety of reasons – are not capable or willing to exercise autonomy (Olthuis et al. 2012, 2014; van Nistelrooij et al. 2017).

Additionally, although many people may like the idea of non-treatment options (such as a do-not-resuscitate order, for instance), when it is still a future anticipation, research shows that most people, when confronted with a serious (physical) crisis, tend to prioritise other treatment possibilities rather than preparing for death (Murray and Amblàs 2021). Hence, despite all the attention for ACP, resuscitative efforts and curative care interventions continue to increase in the last months of life (Murray and Amblàs 2021). With too strong a focus on choice and decision-making we may thus run the risk of overlooking (and misunderstanding) the fact that, when it comes down to it, the majority of people tend to seek medical rescue rather than face the reality of an imminent death. Basically, dealing with end-of-life choices is often experienced as a confrontation with one’s mortality and as such as a deeply existential affair (Callahan 2011; Gawande 2014) that raises the most fundamental doubts and dilemmas among those involved.

To align healthcare and support to these challenges, it is imperative to better understand how these complex choices are lived by those involved. I believe such an understanding is indispensable for patients, their close ones and health professionals alike, as it allows for integration of the lifeworld perspective of the person concerned in end-of-life care in a more substantial way. It may promote opportunities for authentic conversations between people and foster drawing up meaningful ADs. To achieve this, the goal of this paper is twofold. The first aim is to provide a deeper understanding of what it actually means for a human being to make decisions about anticipatory end-of-life treatments or options. How are these complex choices lived? What are the existential implications of anticipating an uncertain and sometimes frightening future? My second aim is to make a significant theoretical contribution to bioethics and ACP-theories by rethinking the concept of end-of-life choices from a phenomenological viewpoint. How can we describe the notion of end-of-life choice in such a way that it reflects the lived experience?

In order to make my case, this paper is divided into three main parts. First, drawing on empirical phenomenological study, I will present a case narrative. While I do not claim that this narrative is typical for all people facing end-of-life choices, I nevertheless believe that it elucidates some of the core characteristics of how these anticipatory choices are lived. Secondly, I will map out a theoretical framework about choice based on the phenomenology of the will of Paul Ricoeur. Finally, guided by this Ricoeurian framework, I will investigate what choice might mean in the contemporary context of ACP-trajectories: what lessons can be learned that help us to deal better with these kinds of choices?

## Case description

Following the empirical turn in ethics (Rehmann-Sutter et al. 2012; Leget, van Nistelrooij & Visse, 2017), I start by presenting a narrative case that draws on recent empirical work (Antonides and van Wijngaarden 2024). The overall goal of this empirical phenomenological study was to articulate the lived experience of relatively healthy older adults who are engaged in a process of anticipating end-of-life choices that may conceivably need to be made in the year(s) to come. The aim was to describe how the choice-making processes were lived (Antonides and van Wijngaarden 2024). Ten triads, each consisting of 1 older adult and 2 nominated close ones, participated in the study (in total *n* = 30). The older adults (age 75–88 years) were all actively engaged in explicating choices regarding their own death and dying in one way or another. All participants were interviewed three times over a period of 2 years (For more detail see: Antonides and van Wijngaarden 2024). The findings of this study indicate that the meaning structure of anticipating end-of-life choices could be understood as a continuous, dynamic and relationally entangled navigation between paradoxical choices regarding an uncertain future (Antonides and van Wijngaarden 2024). The case below draws on one triad, consisting of an older lady, her son and her daughter. It is based on a set of interviews and written by the author for research goals. In order to provide in-depth insight into their lived experience, the case is described quite extensively. To strike a balance between protecting the privacy of the participants and providing sufficient detail needed for a lived experience description, some specific, nonessential information was disguised (van Wijngaarden et al. 2016).

### Existential gambling

Consider the following case: Anne is an older adult (79) who highly values recording her end-of-life choices in advance. It suits her, she states. She always has had a very practical approach towards life. During her working life, she had been a social worker and in that role she had held many conversations with people about important moments in life, including what it means to live towards the end-of-life. She likes to take time for reflection. A few years ago, after the death of her husband Ben, she started drawing up a document for herself in which she notes how she envisions her own end-of-life.

*Just before Ben passed away*,* he spent a month in the ICU. He was 71 at that time. Really*,* it was a terrible situation*,* for him*,* but also for me. I sat there*,* next to his bed*,* day after day*,* watching to see if he would wake up. He was kept in a kind of sleep state. The moments he regained consciousness were the most difficult for me. He was mumbling regularly in his delirium*,* he hardly knew where he was. [With a deep sigh: ] And the pain*,* the pain… I felt so powerless.*

*Afterwards*,* he spent another month in a rehabilitation centre. But then*,* all of a sudden*,* his situation got worse again. They immediately took him to a university hospital for emergency surgery. I wasn’t there in time. He was already on the operating table when I arrived*,* I never spoke to him again… On arrival*,* we were told by the operating surgeon: ‘Your husband really wanted to have the operation’. For us that could only mean that he still wanted to fight*,* he still wanted to live*,* he was prepared to go through all that misery again. For himself and for us.*

*The operation went well*,* but immediately afterwards he went into cardiac arrest. We were sitting in the family room when a doctor abruptly came in and said: you have to come now*,* we are resuscitating. For a moment*,* I was shocked*,* because previously Ben had clearly indicated that he didn’t want to be resuscitated anymore. At two distinct moments*,* a doctor had asked him very explicitly: “Do you want to be resuscitated or not?” And twice he had answered very firmly: “No*,* I don’t want to be resuscitated.” But apparently his wishes were not known in this hospital. So we all ran in there and I shouted: ‘Stop*,* he really didn’t want this.’ After they stopped*,* he died around ten o’clock that evening.*

*To be honest*,* afterwards I struggled with my decision. Because I felt I was the one who had stopped Ben’s resuscitation. Fortunately*,* we had agreed on it together earlier*,* I’m backed up in that sense. Nevertheless*,* I often think to myself: What if I had let them do their job? Also*,* the fact that he really wanted to have another surgery while he was still recovering from the previous operation… It makes me think: oh*,* gosh*,* what if his ideas about resuscitation had changed as well. I just don’t know…*.

*And then I ask myself*,* how much time did we actually spend on his comment? He said “I don’t want to be resuscitated*,*” and that was it. Conversation over. But what if I had asked: what exactly do you mean by that? In which situation? Because it may be very different when you suffer a cardiac arrest here [at home] or in the hospital. Then your chances are much higher… Other times*,* I wonder: when I shouted at those doctors to stop*,* was I really concerned about him? Or was I just trying to keep some form of control in this chaotic situation? To me*,* that is a big difference. I am still not clear about that. And maybe the most difficult thought that sometimes crosses my mind is: what did my children think of the fact that it was me who shouted ‘stop’…*.

*Anyway*,* I’ve started to think a little differently about resuscitation…*

*The experiences also made me think: what do I want for myself? Do I want to go to an ICU*,* for example? And do I want to be resuscitated? So meanwhile I have completed my own list with end-of-life wishes. [She reads from her wish list: ] Not to be transported to the hospital if the home situation allows me to be cared for at home. If admission to hospital turns out to be unavoidable*,* do not use a ventilator or artificial feeding. No tube*,* no antibiotics*,* no chemo*,* no participation in experimental treatments. And in case I get dementia*,* I would want a doctor to perform euthanasia. I emailed the document to my son and daughter. Just so that ‘everything is documented and arranged’. Now they know what I want and they can take care of it.*

*Yes*,* in a way*,* it is a strange enterprise. On the one hand*,* when I wrote it down*,* it felt as if some power was being mobilised within me. I sort of woke up completely. But at the same time*,* it’s a very weird document. Basically*,* it is a theoretical exercise. Because I’m not in that situation right now. So I am sitting here*,* behind my desk*,* anticipating my future*,* writing out all kinds of scenarios: suppose this*,* suppose that…*.

*You know*,* having first-hand experience*,* of course I am deeply convinced that you actually can’t imagine what it would be like in such situations [sighs]…. At the ultimate moment*,* things always turn out differently than expected or planned. Your preferences and choices shift and change over time. Sometimes there is simply nothing to choose*,* instead life just happens to you. And what you really didn’t want*,* turns out to happen anyway. Even if you get the chance to be involved in medical choices*,* you often don’t know exactly what is best*,* you simply lack the knowledge. And last but not least*,* your choices are clouded by the fact that ultimately*,* as human beings*,* we tend to hang on to life. It is so unpredictable*,* so unclear*,* so uncertain. In a way it is a form of existential gambling. Sometimes I wonder: Can you actually say anything meaningful about it if everything is so situationally determined…? And yet I value writing it down…*.

The case above illustrates several problematics mentioned in the introduction. It shows that anticipating end-of-life choices is highly difficult. While in theory, the choices or preferences at stake might be quite clear, defined and reasonable, due to the challenges of the illness trajectories, in practice exercising choices turns out to be much more complex. In fact, the choices are surrounded by fear and uncertainty. Confronted with another crisis, rather than preparing for death, Ben prioritised an additional surgical treatment. Not only does the case show how difficult it is for patients to anticipate their own preferences and choices, it also highlights another difficulty, namely the interpretation of other people’s choices: how should Anne interpret Ben’s choices? Even though she talked them through with Ben in advance, Anne voiced doubts about whether she made the right moral decision at the supreme moment. Notwithstanding the fact that Anne finds she is still puzzled by open-ended questions, in the end, she also remains convinced that proactively anticipating the end-of-life has value for her. Later on in this paper, drawing on Ricoeur’s phenomenology of the will, I will come back to the case and reflect on the lived complexity of end-of-life choices in more detail. But first, let me provide a rationale why Ricoeur’s work fits my purpose. Then I will map out some of the main notions from his theory that I consider important building blocks for rethinking end-of-life choices and describing them in such a way that our descriptions better align with the lived experience.

## Why Ricoeur?

In 1950, Paul Ricoeur – a French philosopher – published *Freedom and Nature: The Voluntary and the Involuntary*. This work is considered to be Ricoeur’s first own philosophical study. In *Freedom and Nature*, Ricoeur develops a phenomenological description of the essential structures of our being-in-the-world, and more specifically of the human will. Ricoeur explores the question: ‘What does it mean for a human being to will, to choose, to decide?’ In his *Intellectual Autobiography*, Ricoeur himself calls this early work ‘a sort of philosophical anthropology’ (Ricoeur 1995, p. 12). Deeply grounded in the lived realities, he attempts to unravel how choice is actually performed by human beings.

In his philosophy of the will, Ricoeur discusses the common topics of free will and determinism, mind and body, freedom and fate. But in contrast to classical philosophical traditions that tend to assume a dualism between freedom and fate, Ricoeur’s guiding principle is their reciprocity (Kohák, 1966, p. xxii). He constantly aims to integrate the encompassing dialectic of mastery and consent, acting and undergoing, activity and passivity, the voluntary and the involuntary (Ricoeur 1995, p. 12). On the one hand, he views the human being as a subject who is master of himself, capable of placing desires and internal and external powers at a distance. On the other hand, he perceives the human being as a servant of necessity. Moreover, with his conception of ‘the reciprocity of the voluntary and the involuntary’, Ricoeur aims to keep these two poles in a dialectic tension rather than synthesising them. As such, Ricoeur not only rejects a view of human beings that loses sight of freedom altogether, thereby reducing people to passive victims of a radically alien nature. He also rejects the assertion of a pure, free will which refuses or trivialises nature. It is exactly this dialectic understanding which makes Ricoeur’s early philosophy of the will a unique lens for inquiry into the lived reality of end-of-life choice-making processes.

Altogether, Ricoeur’s phenomenology offers a suitable focus on the lived experience of choice-making processes that complements the current focus in (bio)ethical studies on ‘reason’ as the central tenet of choice-making, and principled discussions about the value of autonomy and individual decision-making capacities (Beauchamp and Childress 2009). Secondly, it helps to move beyond the customary, dualistic understanding that tends to make a distinction between agency, autonomy and self-determination on the one hand *versus* dependency, passivity and determination on the other. Instead, it proposes to view these concepts as inseparable and complementary modes of our being-in-the-world (de Boer 1993, p. 27). As such it provides a starting point to clarify the more fundamental questions about how to navigate (and maintain the tension between) the widespread wish for agency and control at the end of life on the one hand, and the experienced contingency and uncontrollability of (the end of) life on the other. Thirdly, by highlighting that individual selves are not independent choice-makers but can only make sense of themselves (and their choices) in and through continuous involvement with the surrounding world, Ricoeur has developed a decentralised notion of self. Drawing from there, his philosophy probes choice and decisions not in terms of individual behaviour(s), but primarily as relational, socio-culturally embedded issues. Finally, it also illuminates the importance of incorporating the existence and value of ambivalence and hesitation as key elements in our conception of (well-considered) choice-making processes. Let us now explore these aspects of his philosophy in more depth.

## Choice as a self-affirmative act

In Ricoeur’s understanding of the human will, it is by means of willing, deciding, choosing and doing that human beings affirm their identity. We define ourselves (at least partly) in terms of the things we decide. That is, in choosing, we make up our minds, we commit ourselves or we bind ourselves to a certain ‘project’ (Ricoeur 1966, p. 42) by which he refers to what people are engaged in or care about (e.g., a task, an action), having a personal concern for something (Ricoeur 1995, p. 32). In choosing, we recognise a project as our own (Ricoeur, 1966, p. 63). This means that the very act of deciding can be conceived of as a central and constitutive act of one’s mode of being (Ricoeur 1966, p. 42). We initiate a way of being: this is my way (Ricoeur 1966, pp. 63–64).

For Ricoeur, the will contains an action to be done in accord with one’s abilities (Ricoeur 1966, p. 43). For a decision to become real, there should be a capacity for doing, for actually performing the action. Indeed, Ricoeur sees decision and action as continuous: a genuine decision entails the possibility of action, and is fulfilled by it. In a decision, Ricoeur states, we affirm a position with regard to a project: this is how it should be. Moreover, in a decision we also take position in relation to our own action: the project is to be done by me. Accordingly, human beings are expected to take responsibility for the decision and the action that follows from it. Ricoeur thus establishes a close link between deciding and taking responsibility for that decision and the consequent action (Ricoeur 1966, p. 63). To be responsible means to step forward and to take charge of the job to be done. The feeling of responsibility can be viewed both as a form of commitment and as a form of self-affirmation (Ricoeur 1966, p. 58).

Action is precisely the criterion that distinguishes a decision from, for instance, a wish, a preference, a request or a command, Ricoeur underlines. Because in case of either a wish or a command, the thing to be done does not necessarily imply a personal action, but may depend on the course of things or the action of another person (Ricoeur 1966, p. 46). According to Ricoeur, a decision implies that the execution of the project is accompanied at least by *“the capability of action of its author to realise it”* (Ricoeur 1966, p. 40). A decision may be separated from any corporal execution in time, Ricoeur acknowledges, yet it is in action that we put our intentions to the test and show whether it concerns an authentic decision or it turns out to be more of a wish (Ricoeur 1966, pp. 201–202). Consequently, if the act depends on another person, the request for help or support itself can be conceived as one’s own decision and responsibility. Regarding the requested act, however, it may be more appropriate to speak of wishes and, as such, to recognise that it is the other – who is supposed to act – who has to choose and decide and thus bears the responsibility. Hence (end-of-life) choices that are dependent on the act(s) of another person are probably best considered in terms of convergence of choices, wishes and shared responsibilities.

## The inherent limits of human choice

As described above, choice is to be understood as an identity-affirming act in which human beings can express themselves freely. For a full understanding of choice, however, it is important to also fully acknowledge the limitations of choice. That is, human choice is also principally bounded and determined by the realms of the involuntary, including birth, body, character, history and the social world. Ricoeur poses that birth may be the most telling illustration of this boundedness. From the very beginning of our lives, as he puts it, this central event casts a shadow on our freedom: we were thrown into the world before we were able to posit any act voluntarily. Existence was simply imposed on us (Ricoeur 1966, pp. 433).

Ricoeur mentions that birth confronts us with the realisation that life is utterly contingent: it occurs by a combination of chance, instinct, two given parents, a particular combination of genes and chromosomes, among many other things (Ricoeur 1966, p. 435). Basically, we appear as a random combination (out of a considerable number of combinations which did not come about), which makes us realise that in essence we are determined, contingent beings. In a way, this also implies that from the very beginning of human life, there is no question of free choice. Ricoeur notes, however, that this should not be confused with absolute dependence or total lack of freedom. Instead, he makes a plea for a more dialectic understanding: Humans are not only essentially connected with others and part of a lineage. Also, birth is the beginning of a unique human being, the beginning of an ‘I’ with its own agency. Notwithstanding an already determined starting position, people can determine a direction or an attitude within that given situation (on which I will elaborate a little more below).

Another limitation of choice, underlined by Ricoeur, is that human freedom to choose should be perceived explicitly as an incarnate, embodied freedom (Kohák, 1966, p. xxxiii). Each human body has its own autonomy, its own intentions and its own initiative beyond our will: We have nothing to do with many bodily aspects such as the beating of our heart and the body’s metabolism. We are highly dependent on our body to carry us from infancy to old age. In growing, healing and resting, our body has a nourishing, sustaining, benevolent and protective power. But while our body may be supportive, at the same time, for instance in times of illness, it can also become a threat and even deceptive (Ricoeur 1966, pp. 271, 445). In essence, the human body is ambiguous: it has a will of its own, imposing an indetermination on human choice; a corporal involuntary. Simultaneously, though, the body creates the possibility of being and acting. It is on us to decide to care for, and guide our bodies. Likewise, the human character is ambiguous: on the one hand it can be seen as a form of fate, a set of given traits which identify and typify our behaviour. Yet, Ricoeur argues, there also remains a freedom through which we can mould our character by means of our capacities, effort and decisions (Ricoeur 1966, pp. 366–370).

Yet another restraint on human choice is that choices are always socially and historically situated. Although in *Freedom and Nature* Ricoeur writes more extensively about how the individual consciousness is limited by bodily and more individual aspects, he also explicitly widens the scope to include the historical and socio-cultural embeddedness of the human will. He notes that just like organic life (birth, the body and character), history, society and culture are also at the root of the involuntary as well as the voluntary. As we have not chosen our birth and our bodies, he argues, so we have also not chosen our history or the society and the cultural situation we find ourselves in. Ricoeur explicates, for instance, how society acts out its role in the individual consciousness (Ricoeur 1966, p. 123). Collective representations and social imaginations penetrate the individual human being, and as such they become part of the individual consciousness and form a source of motives driving the choices we make. However, Ricoeur simultaneously underlines that we should not understand the will as completely determined by collective representations, as this would make us miss the essential moment of willing. On the one hand, there are, indeed, the forces of collective, social representations, but on the other hand, he states, there is the autonomy of consciousness. Human beings are able to critically consider social imperatives. Eventually, it is the ‘I’ who evaluates, compares and decides (Ricoeur 1966, p. 125).

While Ricoeur’s philosophy of the human being is often viewed as critical towards the notions of autonomy and agency, he in fact merely nuances the idea of autonomy and autonomous choice, delineating its limits, rather than discarding it, by arguing that autonomy and choice should be understood as phenomena that are deeply embedded in life, and as such, limited and indefinite. By doing so, he advocates a notion of freedom and autonomy, but always understood as embodied and situated. Applied to end-of-life choices, this highlights the need to acknowledge that sometimes, in certain situations, there may be very limited or even no choice. An ill body may simply impose its way on people. A focus on choice-talk would overlook (or even deny) this difficult experience.

## How choices are actually performed

### Consenting as a form of agency

As touched upon above, in a Ricoeurian line of thinking, choosing means acting upon one’s choice. As it is in the acting that we take responsibility for our decisions. As such, we not only commit ourselves to the choice made, but we also affirm ourselves by it. Ricoeur adds, however, that the will is not only related to action as such, but that a full description of the will needs another trait: consent. He includes this notion of consent because the human will also consists of acquiescence (or agreement) to life’s necessities in the form of organic life, birth, body, character, history and the social world. Although the human will can neither propose nor change such necessities (Ricoeur 1966, p. 7), consent makes it possible to understand these involuntary aspects of life in terms of the will (Ricoeur 1966, p. 8). That is, we may be able to say ‘yes’ to a situation which is already determined and which we find ourselves involved in. By consenting we grant our approval to what is already given (Ricoeur 1966, p. 344). Rather than a passive acknowledgement of necessity, Ricoeur understands it as an active decision to accept the situation as one’s own. It is in this consenting act that a human being (re)gains control over the situation (Ricoeur 1966, p. 93). For Ricoeur, consent thus refers to the ultimate reconciliation of freedom and nature: the dialectic of the voluntary and the involuntary. It is one’s freedom which – by means of consent – transforms this given situation into something meaningful that one decides to view as being one’s own (Kohák, 1966, p. xix). Accordingly, it follows that consenting to a given situation, which in end-of-life contexts often seems the only option, can also be interpreted as an autonomous choice. Explicitly recognising this could empower patients and their close ones.

### Socialising one’s needs as a form of agency

By exploring the role of motives in human decision-making, Ricoeur again highlights that the involuntary is always part of the human lifeworld. Because human beings decide not only *in order to*, but also *because of*. The will always has its reasons (including needs, lacks and desires). But he typically states that the reasons that motivate human decisions – bodily, personal and historical – should not simply be conceived as external limitations imposed upon a person (Ricoeur 1966, p. 66). Indeed, a person can choose to appropriate these (involuntary) motives as the motive for one’s own decision (Kohák, 1966, p. xix). In this line of thought, ‘lived needs’, such as hunger and thirst, should also not be viewed merely as an automatic reflex beyond one’s will. Ricoeur states: *“I am not given over to my body*,* I am a self who can take a stand*,* and evaluate my life.”* (Ricoeur 1966, p. 93) He argues that human beings are human beings because of their ability to confront their needs and choose to listen to them, but other times reject or sometimes even sacrifice them. Ricoeur calls this a form of ‘socialising’ one’s needs. It is in this socialising act that one (re)gains control over them and shows one’s humanity (Ricoeur 1966, p. 93).

Ricoeur adds an important nuance: if a person is in serious pain or experiencing deep suffering, mastery over the body in terms of suspending or controlling one’s will to act towards nourishment is often beyond reach. An action which follows from serious pain or suffering should instead be understood as what Ricoeur calls ‘a reflex reaction’ (Ricoeur 1966, pp. 106–107). In such cases, one can no longer be held completely responsible for one’s acts, Ricoeur argues, as pain or suffering frustrates, surprises, overtakes, upsets and attacks the will. Accordingly, action becomes re-action (Ricoeur 1966, p. 236).

### Hesitation as a core characteristic of human choice

Ricoeur underlines the radical significance of hesitation for our understanding of the human will. In his view, hesitation is an attempt to try out and think through different options. Hesitation holds choice in suspension (Ricoeur 1966, p. 137). It often also comes with a kind of decision anxiety (Ricoeur 1966, p. 83), arising from an endless reflection about, for instance, the many possibilities associated with a future project or action. As such, hesitation can have a huge impact on one’s sense of self. Uncertainty about one’s future direction and enduring ‘the mode of maybe’ can be hard or painful. Doubt with respect to a future project can even be experienced as doubt with respect to oneself: we do not know who or what we shall be in the future.

Without trivialising the uncertainty that may be raised by indecision and hesitation, Ricoeur does not see hesitation as a form of weakness or shortcoming. Rather, he argues that the postponement of choice might be understood as ‘a powerful refusal of a naïve belief in an overriding clarity’ (Ricoeur 1966, p. 138). Since human life is utterly complex, consisting of many discordant values and demands, hesitating should be viewed as an acknowledgement of this essential ambiguity of life. Hesitation is also a recognition of one’s own conflicts of values and duties. We have to navigate multiple and sometimes contradictory claims, obligations, pressures, and appeals. It is this plurality of goods and bads that is at the root of hesitation and choice. In addition, a comparison of values is always dynamic: new points of view could always be considered. We will always have an incomplete picture, which makes each hierarchy of values precarious, indefinite and thus questionable (Ricoeur 1966, p. 146).

People hesitate precisely because the world is hugely complex. By postponing, hesitating and inner deliberation we acknowledge the multi-layered, complex nature of our tentative projects and our choices (Ricoeur 1966, pp. 139, 142). This multiplicity, however, is not to be considered an endpoint. Eventually *“choice has to be won from our hesitating consciousness”* (Ricoeur 1966, p. 143). In our minds, we may be able to synthesise viewpoints and leave room for ‘both/and’ but, as Ricoeur points out, when it comes to action, in the end we are usually doomed to separate, sacrifice points of view in favour of others, and just choose ‘either/or’ (Ricoeur 1966, p. 147). Applying these Ricoeurian ideas to the context of end-of-life choices, on the one hand illuminates the radical significance of taking hesitation seriously. Rather than viewing it as a weakness due to having confused reasons, we should make room for hesitation and integrate it as a core aspect of our conception of end-of-life choice-making processes. Nevertheless, Ricoeur’s work simultaneously points to the importance of not trivialising the potential self-affirmative power in overcoming hesitation by choosing. Supporting people in this regard can be seen as equally important.

### Anticipating future contingencies and fears

Another important trait of choosing to be considered is its reference to the future. Most often, people decide for a time to come. To decide is to anticipate. Based on, for instance, previous experiences, knowledge, images, desires, fears and doubts, we have to imagine what the future will be like. In order to decide, to draw up a balance sheet of needs and desires, we have to have some sense of an outline of the future situation. The future, however, is determined by a great number of acts and events that are beyond our power and imagination. It cannot be predicted, at least not with great accuracy. Our future ‘knowledge’, desires or fears represent only an *expectation* of the future. We may weigh the expected future as a threat or a blessing which will *probably* injure or comfort us. This implies, however, that we do not foresee the future, only an expected future (Ricoeur 1966, p. 50).

Sometimes a decision is made just before its execution. Other times a delay separates the decision from its execution. Ricoeur comprehends such deciding in advance as a form of *“human purposiveness which organises time ahead of present.”* (Ricoeur 1966, p. 49) It is an intended decision. People may report that such intended decisions provide a sense of control: by means of advance decision-making they aim to get some form of grip on the future. Eventually, however, such intended decisions, which focus on a more distant future, are often characterised by a sense of ambiguity and uncontrollability. The future, and especially the future further ahead, cannot be controlled. One can attempt to anticipate the future, but at the end of the day, one can only meet it and surrender oneself to it. The voluntary aspects of anticipating thus is not in controlling, but in surrendering.

A specific form of anticipation of the future, which is highly relevant for our inquiry into end-of-life choices, is anticipated pain and suffering. How should we understand the impact of such imaginative anticipations of suffering on the will? First of all, Ricoeur makes us realise that to be afraid of future suffering is to imagine it affectively in the present. As Ricoeur puts it: *“In imagining vividly a stab*,* a burn*,* a bite*,* I affectively see the pain through a present feeling which can spread out and swell into an impelling visceral emotion.”* This means that an image can endow our anticipation with what Ricoeur calls “a quasi-presence” (Ricoeur 1966, p. 258). This way, anticipated suffering may already rule the human flesh before becoming a real-life experience: *“It develops an anxiety which can even reach the level of terror preceding the real bodily experiences of suffering.”* (Ricoeur 1966, p. 107). The human will that is faced with such ‘fear of suffering’ may subsequently try to move away from the imagined painful encounter by, for instance, fleeing, hiding, avoiding or fighting. As such, Ricoeur’s philosophy points to the importance of recognising the realness and intensity of anticipated suffering in the context of end-of-life care. That which is feared may never become true as such, but the imaginary makes it a real lived experience of dreadfulness (de Lange 2014; Ricoeur, 2007/2009, p. 30).

Anticipated, imagined pain or suffering thus stimulates genuine impulses of fear appealing to the will in the same way as needs (Ricoeur 1966, p. 237). But here, in a similar vein as discussed above under ‘needs’, these fears can also be conquered by the will, as the body is not just a bundle of reflexes (Ricoeur 1966, p. 108). While one’s imagination plays an important role in the creation of the feared anticipations, it can also play a fundamental role in overcoming these fears and in the creation of decision. That is, by means of imagination and values, a human being may be able to socialise and master these anticipated fears. Indeed, for Ricoeur the essence of what he calls ‘human wisdom concerning pain and suffering’ does not lie in the repression of its reflexes, but in *“the courage of acting in spite of pain and suffering”* which, in some cases, people have to undergo (Ricoeur 1966, p. 237).

### Self-determination and determination as intertwined realms

Being grounded in a philosophical anthropology (de Boer 1993) that considers the dimension of the involuntary in a more substantial way than is generally the case in contemporary bioethics, I argue that the phenomenology of Ricoeur provides important clues to align our end-of-life decision-making strategies with the real-life needs of those involved. It is helpful to understand these processes as being embedded in historical, socio-cultural and relational contexts. It also illuminates the importance of acknowledging the active and passive aspects of choice-making processes. Human beings are ‘agents’ as well as ‘undergoers’; life is alternately carried out and undergone (Ricoeur 1966, pp. 150, 152, 164). This leads to the Ricoeurian paradoxical understanding of freedom and choice: in choosing, self-determination and determination are linked together (Ricoeur 1966, p. 78; 1995, p. 12); they are two sides of the same coin. Determining oneself means determining one’s gesture in the given world. (Ricoeur 1966, p. 63). It is in this pre-given, determined world that a free human being acts and manipulates, and as such, can be viewed as an ‘author of events’ (Ricoeur 1966, pp. 84, 205). Freedom and fate should thus not be understood as two juxtaposed realms, but rather in a paradoxical, dialectic tension (Ricoeur 1995, p. 12).

## A truly human understanding of choosing and deciding

Having described some of the notions fundamental to Ricoeur’s phenomenological conception of the human will, I now aim to explicate how this thinking can help us to reconsider the notions of end-of-life choices and ACP in such a way that they better align with the lived experience. For this step I revisit the case of Anne and Ben. Based on these reflections, I highlight five underrated elements that in my view should receive full acknowledgement in our theorisations and practical applications regarding end-of-life decision-making. I argue that more attention for these elements may provide people with a refined understanding and more meaningful language that recognises the challenges and tensions in the context of these end-of-life trajectories.

### Acknowledging the passive side of choice

As illustrated in the earlier described case of Anne, given the contingencies of her husband’s illness, his admission to the ICU and his sudden cardiac arrest, she felt that her opportunities to ‘make choices’ were very limited. No matter what Anne and her husband had decided before and had written down in his living will, she felt completely overwhelmed by a situation in which she suddenly found herself. In the terms of Ricoeur, she was an undergoer and a sufferer who was about to lose her loved one. In a split second, in the midst of an emotional roller coaster, she had to make an ultimate decision based on fairly general agreements that she and Ben previously made. Although she did choose to say ‘stop’, in hindsight it did not really feel like a choice to her. In fact, she felt there really wasn’t anything to choose. In her view she could only make a ‘negative choice’. While being an undergoer, Anne at the same time felt called, or almost forced, by the situation, to act. Rather than the one with the most preferable end from several options, she had to choose, or guess, what was probably the least bad option, and as such it would always feel as a somewhat forced, negative choice. Moreover, stopping resuscitation is usually not chosen as an end in itself, but for other reasons, for instance to avoid further suffering. Her situation clearly illustrates that the notion of preference or choice tends to fall short in the complex situations of challenging illness trajectories.

### Acknowledging the value of hesitation

The case of Anne and Ben also shows the importance of viewing hesitation not as a form of weakness or a shortcoming, but rather as a normal human condition, and an acknowledgement of the essential ambiguity of life. Although they previously discussed his do-not-resuscitate order and came to an agreement, this did not guarantee clarity at the crucial moment. Instead, it turned out to be very difficult for Anne to interpret the meaning of Ben’s choices, not only with respect to his written directive, but also regarding the somewhat unexpected wishes that he had expressed that very morning. By her (ongoing) inner deliberation whether she did the right thing or not, she acknowledged the multi-layered, complex or even problematic nature of end-of-life choices. Rather than try to overcome it and solve it, it would probably be better for her to live with and through this hesitation. As such she may be able to start to make some sense of the reality of her life. Anne’s story also shows that hesitating about her own preferences and choices regarding her own end-of-life – and not attaching herself to one of the options yet – is anything but a flaw. Instead it can be seen as a testimony of what she has experienced in her own life; proof of the wisdom she has acquired. At the same time, it demonstrates that – despite what she went through – she still believes in the importance of making an advance directive herself. Putting her own wishes on paper arouses a sense of “power” and “awareness” in her, as she put it. For her, the value of an AD probably does not really lie in listing the precise types of choices that should be made, but rather in the fact that the practice of AD as such is experienced as an affirmation of her agency: it is the process (rather than the directive itself) that creates a space to explicate her values and fears with regard to the end of life.

### Acknowledging the realness of anticipated suffering

Anne’s story also draws our attention to the significance of temporality for our understanding of how choice is lived. It shows how the past and the future influence the present day as interconnected spheres (Todres et al. 2007). Anne’s past strongly impacted her current life, not as factual occurrences but as meanings in the form of memories, reliefs, regrets and doubts. The past events –such as the disease trajectory, the long, traumatic stay in the ICU, the abrupt termination of the resuscitation of Ben– and the associated images and fears still puzzled her and influence how she deals with current (end-of-life) choices. By making her preferences and choices explicit in advance, she wanted to safeguard herself from anticipated pains and suffering. Rooted in her prior experiences of witnessing the suffering of her beloved husband, Anne vividly experienced this imagined suffering as a current impalpable, dangerous threat, already affecting her body and senses in the here-and-now. Writing down intended (not yet to be carried out) decisions functioned as a way to deal with these anticipated fears, and notwithstanding the experienced insecurities, it also provided her with some sense of agency and grip on her situation.

### Acknowledging that agency is in consenting and surrendering rather than controlling

Ultimately, however, Anne was trying to control a process that was fundamentally beyond her control. While she longed for a sense of agency towards the future, her story indicates very clearly how difficult it is to anticipate the future and to try to control it by deciding in advance. The direction of our end-of-life preferences is guided by expectations and influenced by all kinds of images, desires and fears that might come true or not. Accordingly, it could probably help people like Anne to realise that essentially agency is not about control, but merely about consenting to the uncontrollability of life. Regarding her past, Anne’s agency was not in changing the givens of life, as she will never be able to reverse or revise what has actually happened. There is, however, the option of choosing to consent. When the time comes, she will probably be able to say ‘yes’ to the situation that was already determined and in which she found herself against her will. In a Ricoeurian line of thought, rather than a passive acknowledgement of necessity, this could be seen as an active decision to accept the situation as her own. Consent could even provide her with a sense of ‘authorship’ of her own life story. Regarding her future, agency, rather than in controlling, would lie in socialising her fears, and surrendering herself to life as it unfolds. That is, Anne should give herself the freedom to maintain or adapt and change her motives and plans, instead of pinning herself down by recording specific preferences and scenarios. Only by socialising her fears and surrendering herself to principally unknown situations, she may be able to ‘master’ a process the flux of which will always remain beyond the human grip.

### Acknowledging the importance of co-responsibility

The case of Anne and Ben highlights the importance of the notion of co-responsibility in the context of (anticipatory) end-of-life choices. In consultation with Anne, Ben had personally decided about his do-not-resuscitate request in advance. However, considering this decision only in terms of “his decision” or “his choice” misses the mark. It may result in overlooking the link between deciding something and actually taking responsibility for that decision and the action that follows from it. If the act following from Ben’s request depends on Anne, both should realise and acknowledge that it is Anne who (at least also) bears responsibility, as Ricoeur underlines in his work. In fact, at the decisive moment, she is supposed to commit herself to his earlier request. That is, she is also the one who has to decide and act and, after all, has to come to terms with the decision she had to make. Responsibility thus shifts and becomes shared. Consequently, in cases like these, a probing question is whether it may be appropriate to speak more consequently in terms of wishes (rather than directives, choices or decisions), thereby recognising that it is often another person who is expected to decide and act, and as such has to take on the final responsibility.

The case of Anne and Ben points to an additional issue that illustrates the complexity of taking responsibility for the execution of another person’s advance directive. For Anne, especially in hindsight, it was not crystal-clear that the choice which was made explicit in the past (to not resuscitate) reflected the best option when the time to choose was actually there. The situation and circumstances had changed. Ben had turned out to be a fighter who, against all odds, wanted to live. These developments complicated her process of coming to terms with her decision. She also realised that it probably would have been better to not only talk about the ‘what’ of his wishes, but rather talk more deeply and frequently about ‘why’ and ‘how’ this wish mattered to Ben. Notwithstanding the fact that Anne was deeply aware of the complexity of making choices on behalf of another person and taking (co-)responsibility, she herself seemed to overlook this when documenting her own advance directive. In her directive, she stated that she did not want to end up in the nursing home if she got dementia and that she would prefer to receive euthanasia instead. Anne perceived it as ‘obvious’ that she expected her children to ‘respect’ these ‘choices’ and that they ensured her that these choices would be exercised by them once she herself was no longer able to communicate. It would, however,  probably be more appropriate to speak of wishes and acknowledge the fact that after all, not she herself, but her children and the health professionals involved are the ones who ultimately have to decide. Given the circumstances, and according to their own moral standards, they should consider her wishes and weigh, choose, decide what is best and then take responsibility. It would allow due recognition of the burden of deciding for others, probably making it less complicated for people to take up the responsibility for the end-of-life wishes of their close ones, and choose on behalf of them.

## Conclusion

By providing an in-depth empirical-philosophical exploration of what it actually means for a person to anticipate end-of-life choices, my aim was to illustrate how –in the midst of the complexity of everyday life– choosing and deciding most often occur as choosing between grey and grey rather than between black and white. The inquiry highlights the crucial need to acknowledge the essential ambiguity of life. To do justice to the existential, relational and moral challenges concerned, we need a richer and more refined vocabulary regarding ACP than is currently used in policy documents, manuals and leaflets. Rather than focus predominantly on notions such as choice, control, autonomy, agency, self-affirmation, considerations, decisions, motives, preferences, options, pros and cons, balancing and weighing, and providing (medical) instructions, a comprehensive vocabulary should equally include notions like contingency, ambivalence, hesitation, indecision, anxieties, dependence, passivity, consent, surrendering, values, needs and concerns. Moreover, since end-of-life choices are almost never completely stable and fixed, our vocabulary should foreground the idea of a dynamic process that evolves over time. Only if we proportionally integrate these underrated elements in one vocabulary, will we be able to build a language that reflects the challenges and tensions of real life.

Departing from a phenomenological perspective, I argue that such an enriched, refined and more complete language is urgently needed to promote possibilities for more substantial and meaningful end-of-life conversations between all those involved. Furthermore, while it is probably not realistic to change the widely established terms ‘advance *directives*’ and ‘advance care *planning*’, we should realise that the terminology in itself – advance directives as well as advance care planning – already complicates our understanding of what these practices are basically about: Navigating often highly uncertain life trajectories, the fear of future suffering, dealing with one’s mortality cannot be adequately approached as if they are controllable and manageable issues (Conrad 2007; Illich 2003). While the pursuit of personal choice and autonomy can be considered emancipatory and self-affirmative, an overemphasis on choice, control and planning makes it difficult to face contingency and ambivalence as inevitable constituents of the end of life. Accordingly, people may end up unable to come to terms with (let alone to give meaning to) fragility and mortality as integral parts of life (Wardrope 2015).

This brings me to the question: would advance care ‘wishes’ or ‘aspirations’ not be a much better and more adequate expression? That is, whereas choice, directives and planning have an object, wishes, aspirations, longings involve an orientation. As such, the language of wishes and aspirations would probably better reflect life’s flux. It would stimulate going beyond the ‘what-do-I-want’ talk and (also) talk about ‘why-it-matters-to-me’. In addition, such wording would also do much more justice to the shared responsibilities touched upon in the analysis above. Rather than merely talk about ‘my’ decisions, choices or directives, it would be more adequate to acknowledge - also in the terms we use - that in most cases others (like close ones and health professionals) are involved as well, others who have to make up their own mind, and take their own responsibility, especially if my preference requires an act that is to be (partly) executed by another person. Since language not only describes but also shapes our lifeworld, this is hugely important.
